# Concurrent remission of lymphoma and Sjögren’s disease following anti-CD19 chimeric antigen receptor-T cell therapy for diffuse large B-cell lymphoma: a case report

**DOI:** 10.3389/fimmu.2023.1298815

**Published:** 2023-12-19

**Authors:** Lingshuang Sheng, Yilun Zhang, Qi Song, Xufeng Jiang, Weiguo Cao, Lei Li, Hongmei Yi, Xiangqin Weng, Sheng Chen, Zhongmin Wang, Wen Wu, Li Wang, Weili Zhao, Zixun Yan

**Affiliations:** ^1^ Shanghai Institute of Hematology, State Key Laboratory of Medical Genomics, National Research Center for Translational Medicine at Shanghai, Ruijin Hospital, Shanghai Jiao Tong University School of Medicine, Shanghai, China; ^2^ Department of Radiology, Ruijin Hospital, Shanghai Jiao Tong University School of Medicine, Shanghai, China; ^3^ Department of Nuclear Medicine, Ruijin Hospital, Shanghai Jiao Tong University School of Medicine, Shanghai, China; ^4^ Department of Radiation Oncology, Ruijin Hospital, Shanghai Jiao Tong University School of Medicine, Shanghai, China; ^5^ Department of Critical Care Medicine, Ruijin Hospital, Shanghai Jiao Tong University School of Medicine, Shanghai, China; ^6^ Department of Pathology, Ruijin Hospital, Shanghai Jiao Tong University School of Medicine, Shanghai, China; ^7^ Department of Neurology and Institute of Neurology, Ruijin Hospital, Shanghai Jiao Tong University School of Medicine, Shanghai, China

**Keywords:** CAR-T cell therapy, chimeric T cell receptors, autoimmune diseases, Sjögren’s disease, immunotherapy

## Abstract

Anti-CD19 chimeric antigen receptor (CAR)-T cells not only target CD19-positive malignant lymphoma cells but also normal B cells. The utility of CAR-T cell therapy has been reported in rheumatoid arthritis and systemic lupus erythematosus; however, its use in Sjögren’s disease (SjD) remains unknown. In this study, we describe the case of a 76-year-old woman with active SjD for 10 years who was diagnosed with diffuse large B-cell lymphoma. After receiving anti-CD19 CAR-T cell therapy, she achieved complete remission (CR) on day 28. Since the onset of her 10-year history with SjD, she was negative for antinuclear antibodies and anti-Ro-52 for the first time on day 90 after CAR-T cell therapy. Six months after CAR-T cell therapy, the CR status was maintained, serum cytokine levels returned to their normal levels, and dry mouth symptoms improved. The EULAR Sjögren’s Syndrome Disease Activity Index score decreased from 5 to 2, indicating a partial remission of SjD activity compared with that before CAR-T cell treatment. In the early stage of treatment, she presented with grade 2 cytokine release syndrome and grade 1 neurotoxicity, which were completely controlled after an active intervention. This case highlights the potential application of CAR-T cells in treating autoimmune diseases, such as SjD.

## 1 Introduction

Chimeric antigen receptor (CAR)-T cell therapies targeting CD19 have demonstrated remarkable efficacy in the treatment of relapsed or refractory B-cell malignancies (1). Compared with CD20, CD19 is extensively expressed during the maturation of pro-B cells into plasmablasts, making it an optimal immunotherapeutic target. In autoimmune diseases, the immune system is activated against self-antigens, which leads to the release of autoantibodies from plasmablasts and plasma cells as well as tissue damage by cytotoxic T cells.

CAR-T cell therapy was initially approved by the U.S. Food and Drug Administration in 2017 as a treatment option for refractory pre-B-cell acute lymphoblastic leukemia and diffuse large B-cell lymphoma (DLBCL) (2). In addition to their cytotoxic effects on hematologic tumor cells, CAR-T cells can be engineered for treatment of several other solid tumors (3). CAR-T cells can eliminate abnormally activated B cells in autoimmune diseases (4). Recent studies have reported successful remission of autoimmune diseases, such as systemic lupus erythematosus (SLE) and myasthenia gravis, following CAR-T cell therapy (5–7). Several phase I and II studies on CAR-T or CAR-Treg cell therapy for autoimmune diseases, such as myasthenia gravis, neuromyelitis optica spectrum disorder, SLE, and scleroderma, are still ongoing (8). However, only a few studies have reported the use of CAR-T cell therapy for B-cell-mediated autoimmune diseases, including SLE (9), antisynthetase syndrome (10–12), and systemic sclerosis (13). Sjögren’s disease (SjD) is a prototypical autoimmune disease characterized by the activation and accumulation of B cells in target organs, and patients with SjD are more likely to develop B-cell lymphoma (14). Currently, no disease-modifying drugs have been approved for the treatment of SjD. Several studies have evaluated rituximab for the treatment of SjD; however, limited benefits of rituximab were reported in two large randomized controlled trials (15, 16). There are two ongoing phase I studies (NCT05085431 and NCT05859997) focusing on the safety and efficacy of CD19/BCMA CAR-T cell therapy for SjD. The primary outcomes of these studies are dose-limiting toxicity and incidence of treatment-emergent adverse events.

Herein, we report the case of an old female patient who was diagnosed with DLBCL along with SjD and achieved concurrent complete remission (CR) following anti-CD19 CAR-T cell therapy.

## 2 Case report

A 76-year-old female patient was diagnosed with a nongerminal center B-cell-like subtype of DLBCL. She visited a doctor because of lower abdominal distension and pain, and abdominal B-ultrasonography revealed multiple lymphadenopathies in the posterior peritoneum. Positron emission tomography (PET)–computed tomography (CT) also indicated enlargement of multiple lymph nodes in the posterior peritoneum and peripancreatic regions, with increased metabolic activity. After bone marrow puncture, no bone marrow infiltration was observed. Fluorescence *in situ* hybridization in tumor tissues revealed that the probe for B-cell lymphoma (BCL)-6 was positive, whereas that for BCL-2, C-MYC, IRF4, and TP53 was negative. Second-generation gene sequencing on tissues from the patient was performed, and categorized into different prognostic groups based on genetic mutation characteristics. The DLBCL 114 sequencing panel list is provided in the **Supplementary File 1**. According to the LymphGen algorithm classification (17), her molecular subtype was BN2, and mutations in *BCLAF1*, *BTG2*, *DTX1*, *FAS*, *IGLL5*, *JAK2*, *KRT20*, *PIM1*, *PTPN6*, *P2RY8*, *SPEN*, *UBE2A*, and *ZFP36L1* were detected. Based on the Ann Arbor staging, she had advanced stage IIIA DLBCL with an international prognostic index score of 2. In addition, she reported having suffered from SjD and secondary interstitial pneumonia for over 10 years. Ten years ago, she experienced mouth and eye dryness, accompanied by high levels of circulating autoantibodies (ANA: 1:320; anti-Ro-52: ++). After a labial gland biopsy, according to the 2016 American College of Rheumatology/European League Against Rheumatism (ACR/EULAR) diagnostic criteria, the patient’s lip biopsy showed one focus of lymphocytes per 4mm², anti-Ro-52: ++ positivity, with a cumulative score of 6 points, she was diagnosed with SjD with an EULAR Sjögren’s Syndrome Disease Activity Index (ESSDAI) score of 4 (glandular domain: 1, biological domain: 1, lymphadenopathy: 1, hematological domain: 1) (18). After 6 months of intermittent oral glucocorticoid therapy, autoantibody levels were still abnormal and the symptoms did not improve significantly. Consequently, the patient stopped using the drugs on her own. After 2 years, chest CT revealed interstitial pneumonia and she was administered prednisone (30 mg qd) orally again. Her ESSDAI was 5.0 (increased measure of lung domain with 1 score); however, she stopped taking the drug again on her own after 6 months. Chest CT was re-performed intermittently, which revealed that the interstitial pneumonia had not progressed significantly.

**Figure 1:**
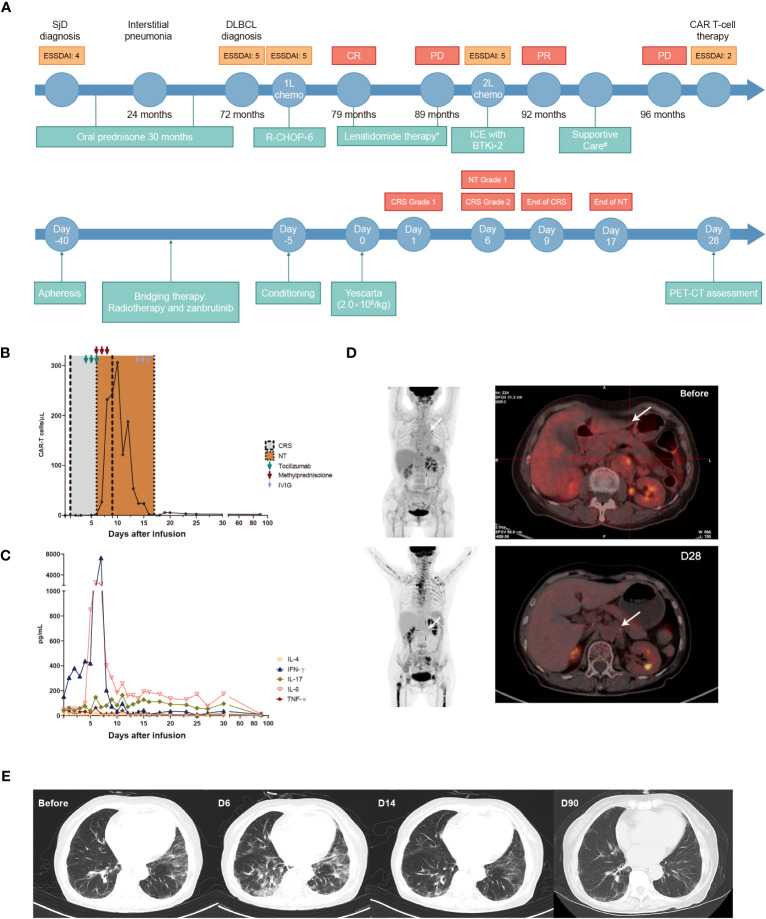
Flow chart of the treatment schedule from the diagnosis of SjD and DLBCL to CAR-T cell therapy. **(A)** Number of circulating CAR-T cells assessed via flow cytometry from day 0 to day 90 after CAR-T cell therapy. **(B)** Changes in the levels of five types of cytokines from day 0 to day 90 after CAR-T cell therapy assessed using a human cytokine detection kit with flow cytometry (Qingdao Raisecare Biotechnology). **(C)** PET–CT images before and 28 days after CAR-T cell infusion. **(D)** Changes in chest CT findings before and after CAR-T cell infusion **(E)**. The white arrow indicates the location of the retroperitoneal lesion. *: Lenalidomide was administered at a dose of 25 mg/day for 1–10 days per month during 79–89 months. ^#^: Supportive care: intermittent transfusion of platelets and other blood products. SjD: Sjögren’s disease; DLBCL, diffuse large B-cell lymphoma; R-CHOP, rituximab plus cyclophosphamide, doxorubicin, vincristine, and prednisone; CR, complete response; PD, progressive disease; PR: partial response; CAR-T, chimeric antigen receptor-T cell; ICE, ifosfamide, carboplatin, and etoposide; IL, interleukin; TNF, tumor necrosis factor; IFN-γ, interferon gamma; IVIG, intravenous immunoglobulin.

**Figure 1A** shows the treatment schedule of the patient. After the confirmation of DLBCL, the patient was suggested six standard courses of first-line rituximab plus cyclophosphamide, doxorubicin, vincristine, and prednisone (R-CHOP) regimen containing 375 mg/m^2^ rituximab on day 0, 750 mg/m^2^ cyclophosphamide on day 1, 50 mg/m^2^ doxorubicin on day 1, 1.4 mg/m^2^ vincristine on day 1, and 60 mg/m^2^ prednisone on days 1–5 of each cycle. The patient accepted the suggestion and achieved CR following treatment, as assessed using PET-CT based on the 2014 Lugano classification (19). Following the first-line R-CHOP regimen, the level of antinuclear antibodies (ANAs) decreased substantially; however, dry mouth symptoms did not improve. Accordingly, oral lenalidomide (25 mg/day, 1–10 days per month) was recommended as maintenance therapy, to which she agreed. Unfortunately, after 10 months, she noticed an enlargement of superficial lymph nodes. PET–CT revealed enlargement of multiple superficial and retroperitoneal lymph nodes along with elevated metabolism. An inguinal lymph node biopsy indicated DLBCL, and recurrence was confirmed. Subsequently, the treatment was switched to second-line ifosfamide, carboplatin, and etoposide (ICE) chemotherapy (1.5 g/m^2^ ifosfamide on days 1–3, carboplatin “area under curve of 5” multiplied by “creatinine clearance rate plus 25” on day 2, and 100 mg/m^2^ etoposide on days 1–3 of each cycle) with Bruton’s tyrosine kinase inhibitor (zanubrutinib 160 mg bid day on days 1–21); this treatment was continued for two cycles. PET–CT performed following two treatment cycles (approximately 3 months of treatment) revealed tumor size reduction and decreased metabolism, indicating partial remission. However, because of thrombocytopenia, she could not tolerate further chemotherapy or targeted agents and stopped taking the medications. She only accepted intermittent blood product transfusion as support therapy. Three months after the second-line therapy, the retroperitoneal tumor size increased from 1.9 × 1.6 cm to 5.3 × 3.1 cm, accompanied by disease progression. The patient’s condition was reevaluated, and her history of autoimmune disease was considered. Meanwhile, her autoantibody levels remained high. CAR-T cell therapy with axicabtagene ciloleucel, a marketed second-line CAR-T therapy containing the CD28 signaling domain, was recommended, which she willingly accepted. After apheresis, she received bridging therapy, comprising retroperitoneal local radiotherapy and zanubrutinib, to lower the tumor burden and expose the tumor antigens (20). Five days before CAR-T cell infusion, the FC regimen (30 mg/m^2^ fludarabine on days 1–3 and 500 mg/m^2^ cyclophosphamide on days 1–3) was initiated. On day 40 after apheresis, she received a CAR-T infusion (2 × 10^6^/kg). Furthermore, blood laboratory tests revealed that she was positive for anti-Ro-52 and ANAs, with a speckled pattern of 1:160 titers as the main fluorescence karyotype. In addition, she showed elevated basal cytokine levels before DLBCL diagnosis. After ruling out the possibility of infection as the cause of elevated cytokine levels, it was suggested that the elevated basal cytokine levels were due to active SjD. Chest CT also revealed a pulmonary interstitial lesion. The patient’s ESSDAI score remained at 5 from the time of DLBCL diagnosis until before CAR-T treatment.

The methods and gating strategy for CAR-T cells are detailed in **Supplementary File 2**. On day 1 after infusion, the patient experienced a series of symptoms, including fever, fatigue, reduced appetite, vomiting, headache, and muscular pain, which were identified as grade 1 cytokine release syndrome (CRS). A potential infection due to the myelosuppression and immune-compromised status of the patient after treatment for lymphodepletion was considered the cause of the fever, and the antibiotic was upgraded from the third-generation cephalosporin to carbapenem. However, this drug could not control the fever, and her body temperature increased to >39°C on day 4. Cytometry assay revealed gradually increasing levels of numerous cytokines along with an abundance of CAR-T cells (**Figures 1B, C**). Accordingly, active CRS was confirmed. To manage CRS, the first dose of tocilizumab (8 mg/kg) was prescribed on the same day (**Figure 1A**); however, her fever remained uncontrolled. On day 5, another dose of tocilizumab was administered. Unfortunately, on day 6, she presented with more symptoms, including CRS-induced facial edema, rales in the lungs, high serum B-type natriuretic peptide levels, and acute heart failure. Emergency chest CT revealed massive exudation in the lungs. In addition, she experienced a hypotensive situation in the absence of vasopressors and was diagnosed with grade 2 CRS according to the American Society for Transplantation and Cellular Therapy consensus (21). Then, the third dose of tocilizumab was administered immediately. Furthermore, the patient presented with limb tremors and decreased writing ability, collectively indicating the onset of grade 1 immune effector cell-associated neurotoxicity syndrome (ICANS). Nevertheless, she still showed clear consciousness and normal memory and calculating abilities. To address her ICANS, methylprednisolone (40 mg bid) was administered on days 6–8. Moreover, levetiracetam (1000 mg bid) was administered to treat seizures. On day 9, her body temperature and blood pressure returned to normal levels and most CRS-related symptoms disappeared. On day 17, her limb tremors also disappeared. In addition, the immunoglobulin levels were within the normal range before CAR-T cell therapy, whereas the immunoglobulin levels decreased after the therapy. After regular intravenous immunoglobulin (IVIG) supplementation (10g/d on days 14–16), the immunoglobulin levels returned to normal levels. Since the infusion, the patient had also presented grade 3 anemia and grade 4 thrombocytopenia, which were treated using granulocyte-colony stimulating factor and thrombopoietin after 1.5 months.

**Figure 2:**
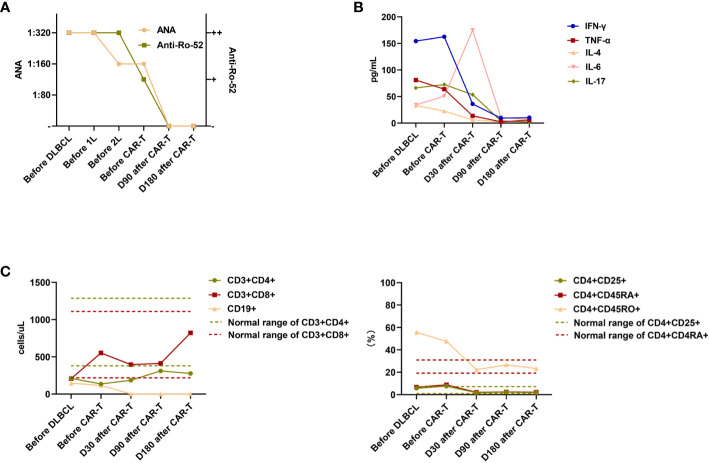
Levels of ANA and Ro-52 **(A)**, cytokines **(B)**, and lymphocyte subsets (including absolute values and percentage values for each lymphocyte subset over all lymphocytes in the patients’ peripheral blood) **(C)** before DLBCL diagnosis; before CAR-T therapy; and on days 30, 90, and 180 after CAR-T therapy. Dashed lines in **(B)** and **(C)** indicate normal ranges of cytokines and lymphocyte subsets. ANA, antinuclear autoantibodies; DLBCL, diffuse large B-cell lymphoma; CAR-T, chimeric antigen receptor-T cell; IL, interleukin; TNF, tumor necrosis factor; IFN-γ, interferon gamma.

On day 28 following CAR-T cell infusion, PET–CT revealed an increase in bone metabolism (standardized uptake value maximum [SUVmax]: 8.5–10.6), possibly due to the use of bone marrow stimulants leading to active bone marrow proliferation. Increased metabolism in the throat region (SUVmax: 7.4) may be indicative of an inflammatory response. The SUVmax of the original retroperitoneal lesion was 1.8–2.0. Deaville’s score was 2, indicating CR (**Figure 1D**). Surprisingly, throughout her 10-year SjD history, she was negative for ANAs and anti-Ro-52 for the first time on day 90 after CAR-T cell infusion (**Figure 2A**). Moreover, significant improvement was observed in the pulmonary interstitium (**Figure 1E**). Approximately 1 month after infusion, cytokines gradually decreased to normal levels prior to CAR-T treatment (**Figure 2B**), which was considered to be partially due to the control of the autoimmune disease by CAR-T treatment (20), although the potential contribution of tocilizumab, methylprednisolone, IVIG treatment, and lymphodepletion cannot be excluded. Her peripheral blood lymphocytes were also monitored, and changes in CD4+, CD8+, and memory Th cells were observed, consistent with the outcomes of SjD management (**Figure 2C**). In most patients with SjD, circulatory CD4+, CD8+, and memory Th cell counts are within the normal range; however, their function may be altered. Currently, the exact influence of different subsets of CD8+ T cells in peripheral blood on the occurrence and progression of SjD is not fully understood. Some studies have suggested that CD8+ T cells play an activating role in autoimmune diseases, whereas other studies have proposed that inhibitory subsets of CD8+ T cells can suppress the infiltration of CD4+ T cells into the glands during the active phase of autoimmune diseases. Activated subsets of CD4+ T cells, particularly Tfh cells, promote disease development by producing proinflammatory cytokines and inducing B-cell activation, thereby establishing a positive feedback loop. Suppressing CD4+ T cells can consequently inhibit abnormal B-cell activation and reduce disease activity (20–22). **Figure 2B** shows the changes in the numbers of lymphocyte subsets during the treatment period, including the marked increase in the number of CD8+ lymphocytes after treatment. Decrease in CD19+ cells might be associated with the control of DLBCL (23). Three months after CAR-T cell therapy, the CR status was maintained, with ANA and anti-Ro-52 levels returning to normal levels. She was satisfied with the significant relief of her dry mouth. The ESSDAI was 2 (lung domain 1 and hematological domain 1), indicating a partial remission of SjD activity.

## 3 Discussion

In this study, we report a case of DLBCL with SjD that showed a significant alleviation of activity after anti-CD19 CAR-T cell therapy. B-cell-activating factor (BAFF), a driving factor for SjD development, significantly contributes to B-cell survival and hyperactivity (24), and several target agents, such as rituximab, a CD20 monoclonal antibody, are based on this rationale for treating patients via B-cell depletion. Similar to CD20 monoclonal antibody therapy, anti-CD19 CAR-T cell therapy could treat SjD via B-cell depletion. In addition, B-cell-depleting antibodies require repeated dosing to maintain B-cell aplasia. Clinical trials have proven that CD19 CAR-T cell therapy, even a single administration, results in more durable B-cell aplasia than CD20 monoclonal antibody therapy (25). Moreover, long-lived plasma cells express none or extremely low levels of CD20 and produce numerous autoantibodies in patients with SjD, which cannot be targeted by a CD20 monoclonal antibody. In patients with SjD, infiltrated B cells and locally differentiated plasma cells reside in the salivary glands, and epithelial cells in this region produce excessive amounts of cytokines and proinflammatory factors for B-cell survival, such as BAFF (24). Compared with CD20, CD19 is extensively expressed during the maturation of pro-B cells into plasmablasts and may be an optimal immunotherapeutic target.

In this case, the patient experienced grade 2 CRS and grade 1 ICANS. ZUMA serial clinical trials have revealed that adverse effects are often associated with high cytokine levels (26–28). Thus, the severe adverse events reported in this case might be associated with the tumor burden and pSS-related high baseline levels of cytokines. Two weeks after CAR-T cell infusion, the patient successfully recovered from CRS, reporting normal cytokine levels. Previous studies have reported CRS and ICANS as adverse events following CAR-T cell therapy in patients with autoimmune diseases (29). Therefore, in patients with concurrent autoimmune diseases, severe CRS or ICANS should be considered before CAR-T cell therapy. In this case, although no direct evidence suggests that autoimmune disease increased the risk of serious adverse reactions to CAR-T cell therapy, the high levels of cytokines indicated that more serious complications of CAR-T cell therapy can occur in patients with autoimmune diseases. Prior to the introduction of CAR-T cell therapy in patients with autoimmune diseases, their basal inflammatory state is induced by inflammatory cytokines produced by infiltration of immune cells, such as M1 macrophages and monocytes, into the lesion tissue (30). Therefore, CAR-T cell therapy should be used with further caution in these patients (31).

This report has several limitations. Despite the high efficacy of CAR-T cell therapy in treating SjD in this patient, this positive outcome might be influenced by several other factors, e.g., tocilizumab treatment on days 4–6 and methylprednisolone treatment on days 6–8. We observed a long-standing 8-month CR of DLBCL after CAR-T cell therapy, along with normal levels of ANA, anti-Ro-52, and cytokines and the improvement of dry mouth symptoms, without the use of glucocorticoids or tocilizumab; in this case, these symptoms had not been well controlled for 10 years. Long-term observation is necessary to confirm the efficacy of CAR-T cell therapy for pSS. Notably, tocilizumab, another possible therapeutic drug for SjD, showed good clinical efficacy in some patients (32–35), but in an RCT reported by Felten et al., tocilizumab did not show a significant effect on SjD (36). Thus, currently, the effect of tocilizumab on SjD remains uncertain. In this study, we only observed a short-term efficacy of tocilizumab; the long-term efficacy and safety of tocilizumab in patients with SjD need to be further evaluated in well-designed clinical trials. Overall, the consistent decrease in antibodies was more likely due to CAR-T cell therapy. CAR-T cell therapy is an effective therapeutic modality for treating B-cell-mediated autoimmune diseases, such as SLE and RA. This case report will provide valuable information for the future application of CAR-T cell therapy in the treatment of SjD.

## Data availability statement

The raw data supporting the conclusions of this article will be made available by the authors, without undue reservation.

## Ethics statement

The studies involving humans were approved by Shanghai Ruijin Hospital Ethics Board. The studies were conducted in accordance with the local legislation and institutional requirements. The participants provided their written informed consent to participate in this study. Written informed consent was obtained from the participant/patient(s) for the publication of this case report.

## Author contributions

LS: Conceptualization, Data curation, Writing – original draft. YZ: Conceptualization, Data curation, Writing – original draft. QS: Conceptualization, Writing – original draft. XJ: Conceptualization, Writing – original draft. WC: Conceptualization, Writing – original draft. LL: Conceptualization, Writing – original draft. HY: Conceptualization, Writing – original draft. XW: Conceptualization, Writing – original draft. SC: Conceptualization, Writing – original draft. ZW: Conceptualization, Writing – original draft. WW: Project administration, Resources, Writing – original draft. LW: Funding acquisition, Project administration, Resources, Writing – original draft. WZ: Funding acquisition, Project administration, Resources, Writing – original draft, Writing – review & editing. ZY: Data curation, Investigation, Writing – original draft, Writing – review & editing.
