# Supplementary material for: Concurrent remission of lymphoma and Sjögren’s disease following anti-CD19 chimeric antigen receptor-T cell therapy for diffuse large B-cell lymphoma: a case report
Source: Front Immunol. 2023 Dec 19;14:1298815. doi: 10.3389/fimmu.2023.1298815 (PMC10762793; doi:10.3389/fimmu.2023.1298815)
Supplement: Supplementary file 1 [file DataSheet_1.docx]

**Supplemental file 1**

The DLBCL 114 sequencing panel list is as follows:

ACTB, ARID1A, ATM, B2M, BCL2, BCL6, BCOR, BIRC3, BRAF, BTG1, BTG2, CARD11, CCND1, CCND2, CCND3, CD36, CD58, CD70, CD79A, CD79B, CHD2, CIC, CIITA, CREBBP, CXCR4, DDX3X, DTX1, DUSP2, EBF1, EGR2, EP300, EZH2, FAS, FBXW7, FGFR3, FOXO1, GNA13, HIST1H1C, HIST1H1E, HIST2H2AB, ID3, IGLL5, IKBKB, IRF4, IRF8, ITPKB, JAK2, KIR2DL1, KLHL6, KMT2C, KMT2D, KRAS, KRT20, LRP1B, LRRN3, LYN, MAP2K1, MAPK7, MED12, MEF2B, MPEG1, MTOR, MYC, MYD88, MYOM2, NCOR2, NFKBIE, NOTCH1, NOTCH2, NOTCH3, NRAS, NRXN3, NSD2, P2RY8, PCLO, PIK3CA, PIK3CD, PIM1, POSTN, POT1, PRDM1, PTPN6, RAG1, RET, RIPK1, ROBO2, SAMHD1, SF3B1, SGK1, ,SIN3A, SLITRK3, SOCS1, SPEN, STAT3, STAT6, TBL1XR1, TCF3, TET2, TGM7, TMSB4X, TNFAIP3, TNFRSF14, TP53, TRAF2, TSC2, TYK2, UBE2A, XPO1, ZFP36L1, ZMYM3, ZNF608

**Supplemental file 2**

The methods and gating strategy for CAR-T cells are as follows. CD19 CAR-expressing T cells were determined using a combination of the following three antibodies included in the CD19 CAR detection reagent: PE-labeled rabbit anti-mouse FMC63 scFv monoclonal antibody (BioSwan Laboratories, Shanghai, China), APC-labeled CD3 (Becton Dickinson, San Jose, CA, USA), and QB500-labeled CD45 (QuantoBio, Tianjin, China). The samples were analyzed using a FACSLyric cytometer (Becton Dickinson) according to the manufacturer’s instructions. At least 10000 lymphocytes with low SSC and high CD45 expression were acquired, and data were analyzed with the KALUZA software (Beckman Coulter, Brea, CA, USA). The CD19 CAR-expressing T cells were defined as those that were double-positive for FMC63 and CD3 in a gated population of lymphocytes and were quantified as the percentage of the total number of white blood cells. The absolute count of CD19 CAR-expressing T cells was calculated by multiplying the white blood cell count with the percentage of the calculated double-positive cells.
